# Discrete Particle Method for Simulating Hypervelocity Impact Phenomena

**DOI:** 10.3390/ma10040379

**Published:** 2017-04-02

**Authors:** Erkai Watson, Martin O. Steinhauser

**Affiliations:** 1 Department of Systems Solutions, Fraunhofer–Institute for High–Speed Dynamics, Ernst–Mach–Institut, EMI, Eckerstrasse 4, 79104 Freiburg, Germany; erkai.watson@emi.fraunhofer.de; 2 Department of Chemistry, Faculty of Science, University of Basel, Klingelbergstrasse 80, CH-4056 Basel, Switzerland

**Keywords:** Discrete Element Method, hypervelocity impact, debris cloud, fragmentation, space debris, multiscale modeling, computer simulation, high performance computing

## Abstract

In this paper, we introduce a computational model for the simulation of hypervelocity impact (HVI) phenomena which is based on the Discrete Element Method (DEM). Our paper constitutes the first application of DEM to the modeling and simulating of impact events for velocities beyond 5 ${\mathrm{kms}}^{-1}$. We present here the results of a systematic numerical study on HVI of solids. For modeling the solids, we use discrete spherical particles that interact with each other via potentials. In our numerical investigations we are particularly interested in the dynamics of material fragmentation upon impact. We model a typical HVI experiment configuration where a sphere strikes a thin plate and investigate the properties of the resulting debris cloud. We provide a quantitative computational analysis of the resulting debris cloud caused by impact and a comprehensive parameter study by varying key parameters of our model. We compare our findings from the simulations with recent HVI experiments performed at our institute. Our findings are that the DEM method leads to very stable, energy–conserving simulations of HVI scenarios that map the experimental setup where a sphere strikes a thin plate at hypervelocity speed. Our chosen interaction model works particularly well in the velocity range where the local stresses caused by impact shock waves markedly exceed the ultimate material strength.

## 1. Introduction

Since the beginning of the space age in the 20th century, the number of man–made debris particles in the Earth’s orbit has constantly risen. Hence, there has been an ever growing risk of active satellites being hit by space debris in the low earth orbit [1,2,3]. In order to asses the risk of future collision events, it is important to be able to predict the impact dynamics of the resulting debris cloud when space debris traveling at high velocity strikes a satellite structure.

The study of hypervelocity impact (HVI) problems is of great interest for many engineering applications, such as shield design for spacecraft protection [4]. The term hypervelocity generally refers to velocities so high that the strength of materials upon impact plays only a minor role and the material ceases to behave as a rigid solid, but rather much more like a fluid [5]. Using the conservation of mass, momentum, and energy, one can make a simplified analysis by neglecting material strength, often referred to as a hydrodynamic model. The velocities at which materials start to behave like a fluid vary widely depending on the material’s shock impedances and can be anywhere between 2 and 10 ${\mathrm{kms}}^{-1}$ [6]. For example, for aluminum, steel and quartz the hypervelocity phenomenon emerges with impact speeds of 5–6 ${\mathrm{kms}}^{-1}$ [7].

**Figure 1:**
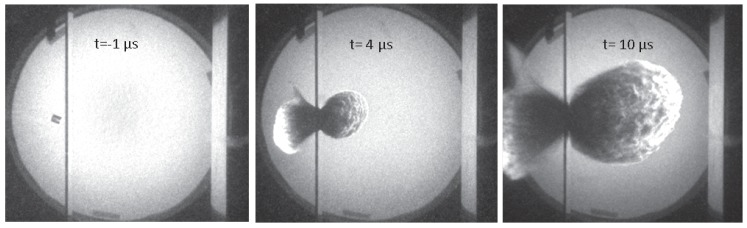
High-speed photograph sequence of HVI performed at Fraunhofer Ernst–Mach–Institut [8]. A cylindrical impactor approaches from the left, collides with the target plate, and the resulting debris cloud propagates to the right.

The typical setup for HVI experiments is that of a small projectile impacting a plate at a $90^{\circ }$ angle. Two important parameters are the impact velocity $v_{0}$ and the ratio of the thickness *t* of the target plate to the diameter *D* of the impactor t/D. Figure 1 shows high-speed camera images from a typical HVI experiment [8]. A polycarbonate cylinder approaches the aluminum target plate from the left with a velocity of 7 ${\mathrm{kms}}^{-1}$. Upon collision, part of the plate and the cylinder fragment into a cloud of debris which continues to expand to the right. The extremely large deformations and material fracture involved in the process of HVI make them challenging tasks for computer simulation.

Continuum based methods are the most widely used technique for simulating HVI. One important method is Smooth Particle Hydrodynamics (SPH) which was originally proposed to solve astrophysical problems in three dimensions [9,10]. It is a meshless continuum method that provides numerical solutions for integral equations or partial differential equations with a set of arbitrarily distributed integration points for the continuum equations of hydrodynamics, called “particles”. SPH has applications in many fields such as astrophysics, hydrodynamics, magnetohydrodynamics, gas explosions, and granular flows, and has also been extended to simulate bodies with material strength [11,12,13,14]. SPH is widely applied to impact problems in computational solid mechanics due to its meshless structure [15,16,17]. Along with Eulerian methods, SPH does not suffer from limitations originating from severe mesh distortions when solving problems with large deformations, in contrast to finite element based methods which require special care in preventing mesh entanglement [18], therefore making SPH an attractive choice for HVI. [19,20,21]. However, SPH does encounter several difficulties in engineering problems such as the tensile instability problem, convergence of the numerical scheme and the difficulty in loading essential boundary condition that may be important for impact phenomena [22]. Although formulations and algorithms have been successfully implemented in SPH which help to overcome some of these limitations [23], the source of the problems still remains.

In an attempt to extend the use of particle methods, hybrid codes, a combination of finite-element and meshfree methods have been developed. Some codes use a combination of FEM and SPH depending on the expected deformations of the specific area [24], while others couple the two methods by converting elements with large distortions into particles [25]. Still others use a hybrid approach where the compressive pressures are computed at the particles and the tensile pressures at the elements [26]. Such codes produce accurate results [27], but contain the combined complexity of both methods. They are often also very specifically tailored to a given setup or problem.

A completely different method for modeling materials undergoing HVI is by using a discrete instead of a continuum approach. A discrete approach approximates a material as a collection of Newtonian particles [28,29]. One such method is the Discrete Element Method (DEM) which originates from Cundall and Strack [30] and has found many new applications in different fields spanning chemical engineering, pharmaceuticals, powder metallurgy, agriculture and many others. Various DEM approaches have been used to simulate the behavior of cohesive granular matter under different impact situations with velocities below 100 ${\mathrm{ms}}^{-1}$. These approaches used differing models and were applied to specific problem areas with varying degree of success [31,32]. To date, DEM simulations have been extensively applied to diverse problems in granular processes such as packing of particles [33,34], flow from a hopper [35,36], die filling [37], fragmentation of agglomerates [38], bulk compression [39,40,41], flow in a screw conveyor [42] and powder mixing [43,44].

While DEM is attracting increasing interest for the simulation of industrial granular flow, much of the previous DEM modeling has considered mostly low–velocity dynamics. In some innovative applications, the DEM method has been applied to shock impact simulations and the fragmentation and failure behavior under shock compression has been investigated [45,46,47]. However, to our knowledge, the potential of the DEM method to be used for hypervelocity impact scenarios beyond 5 ${\mathrm{kms}}^{-1}$ has not yet been explored.

In this paper, we make a novel application of DEM to HVI simulations and explore its suitability for such simulations. We make some simplifications to traditional DEM by neglecting dissipation in the form of friction and damping. Our model’s parameters are chosen to most closely approximate aluminum on aluminum impacts by comparing the simulations with experimental results. We evaluate our model within a wide range of impact conditions to determine its suitability for numerical computation of HVI phenomena.

## 2. Simulation Model

In our simulations, we aim at modeling the dynamics of impact failure and fracture behavior of the material as observed in HVI experiments. For simplicity, we use mono–disperse spheres as basic discrete elements and adjust their interactions using attractive and repulsive potentials. It has been shown that the physical observables determined by such models for granular matter depend mainly on the interaction potentials and much less on the shape of the elements used for the discretization [34,48].

A general principle used to develop our coarse-grained model is to begin with the simplest possible working model before adding more complexity. This simplifies the investigation of the complex interactions between material parameters. As we proceed to show, three parameters appear to be sufficient for reproducing the essential basic material properties that are important in a HVI setting where details of material strength can be simplified due to the overwhelmingly large shock pressures experienced in the case of HVI. The essential properties are, first, the resistance to pressure, second, the cohesive forces that keep the elements together to form a solid, and finally the microscopic failure.

### 2.1. Initial Setup

**Figure 2:**
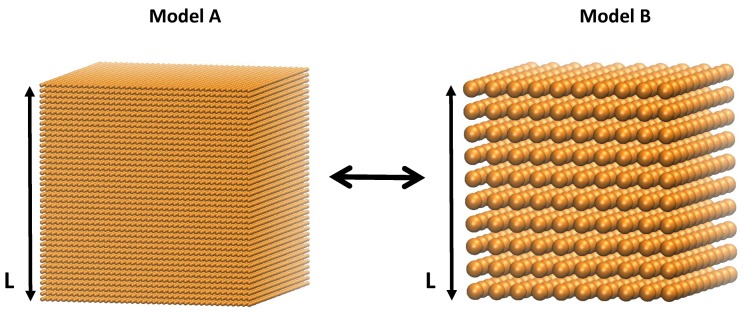
Particles are initiated into a regular cubic lattice. The model’s properties are independent of number of particles.

The particles are initiated into a regular cubic lattice structure, as seen in Figure 2. Each particle has two properties: mass $m_{i}$, and a length scale, diameter $\sigma_{i}$, according to the system’s geometry. In the simulations presented here, we chose a mono–disperse configurations of particles, i.e., all masses $m_{i}=m$ and all lengths scales $\sigma_{i}=\sigma$ are the same for all particles. To form larger solids, many particles are connected with massless spring elements, also referred to as bonds. Then, a small random velocity taken from an equilibrium Boltzmann-distribution is applied to each particle. This random velocity ensures that the load transfer path is distributed through the material by disrupting the perfect alignments of the initial setup.

### 2.2. Particle Potentials

The dynamics of our model is governed by Newton’s second law,

(1) $$\begin{array}{c} -\nabla_{r_{i}}\Phi_{\mathrm{tot}}=F_{i}=m{\ddot{r}}_{i}\;, \end{array}$$

which is used to evaluate the accelerations acting on each particle at every time step during the simulation. $\Phi_{\mathrm{tot}}$ is the total interaction potential, i.e., the sum of all potentials acting on each particle *i* introduced in the next section. The accelerations can then be integrated to yield velocities and positions. The forces acting on each particle are defined via pair potentials. $F_{i}$ comprises the force acting on the *i*–th particle due to the interaction potentials and *m* is the mass of one particle. Interactions can be classified as contact and bonded interactions. Bonded interactions correspond to the pairwise interactions of particles connected by a spring element. Contact interactions are those pairwise interactions experienced by particles whose centers are less than two radius lengths away from each other. We do not consider shear or tangential potentials in this basic model.

#### 2.2.1. Contact Potentials

The Lennard–Jones potential

(2) $$\begin{array}{c} {\phi_{\mathrm{rep}}}^{\mathrm{LJ}}\left(r_{ij}\right)=\varepsilon \left\{{\left(\frac{\sigma }{r_{ij}}\right)}^{12}-{\left(\frac{\sigma }{r_{ij}}\right)}^{6}\right\} \end{array}$$

is a simple potential most commonly used in Molecular Dynamics simulations to model soft spheres [49], where σ is the diameter of each simulation particle, $r_{ij}=\left|r_{j}-r_{i}\right|$ is the distance between two particles, and ε is a pre–factor which has units of energy. The spheres are allowed to interpenetrate each other to a small extent (soft spheres), but quickly experience a strong repulsive potential according to ${\left(\frac{\sigma }{r_{ij}}\right)}^{12}$. Beyond the particle diameter σ, there is a long range attractive component proportional to ${\left(\frac{\sigma }{r_{ij}}\right)}^{6}$. The potential reaches a minimum at $r_{ij}=r_{\min}=2^{1/6}\;\sigma \approx 1.1225\;\sigma$, which defines the equilibrium distance $r_{eq}$.

In the presented model, the Lennard-Jones potential is modified slightly to refine the description of the physics of particle interactions: A cutoff distance, set to the potential minimum, is defined to remove the attractive component. Beyond this distance the potential is defined to be zero. Shortening the potential’s range also provides the benefit of reducing the computational time because each particle interacts with fewer neighboring particles which reduces the complexity of the interaction search algorithm. Additionally, the potential is shifted upwards by the factor ε to ensure smooth continuity with the spring potential such that:

(3) $$\begin{array}{c} \phi_{\mathrm{rep}}\left(r_{ij}\right)=\left\{\begin{array}{cc} \varepsilon \left\{{\left(\frac{\sigma }{r_{ij}}\right)}^{12}-{\left(\frac{\sigma }{r_{ij}}\right)}^{6}+1\right\} & \mathrm{if}\;r_{ij}<r_{eq}\;,\\ 0 & \mathrm{otherwise}\;. \end{array}\right. \end{array}$$

#### 2.2.2. Bonded Potentials

**Figure 3:**
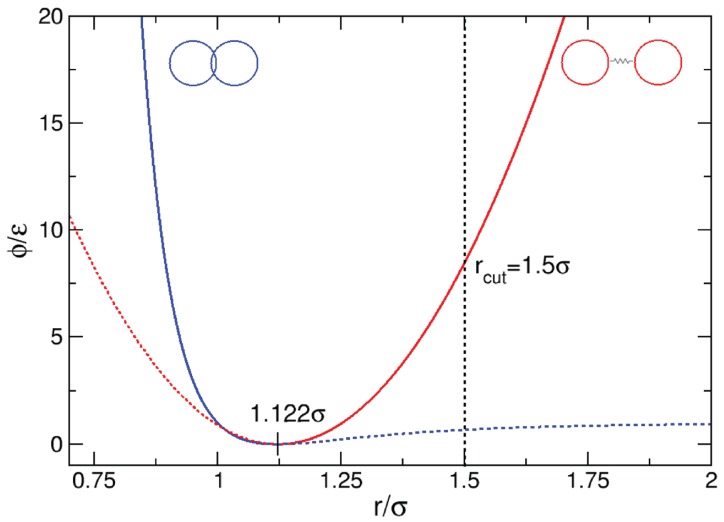
Repulsive and cohesive potentials used in the model. The solid blue line (left side) represents the Lennard-Jones potential and the solid red line (right side) represents the spring potential. The combined blue and red solid lines govern the forces acting on each particle pair; the dotted lines are excluded.

Neighboring particles are linked together to form a crystalline lattice structure. The bonded particle pairs can experience both cohesive and repulsive forces. A quadratic spring potential

(4) $$\begin{array}{c} \phi_{\mathrm{coh}}\left(r_{ij}\right)=\left\{\begin{array}{cc} \frac{1}{2}\kappa {\left(r_{ij}-r_{\mathrm{eq}}\right)}^{2}\; & \mathrm{for}\;r_{ij}>r_{eq}\;,\\ 0 & \mathrm{otherwise} \end{array}\right. \end{array}$$

is used for the cohesive component, and the potential of Equation (3) for the repulsive component. Parameter κ is in essence the spring constant and has units of energy divided by units of length squared. The equilibrium distance $r_{\mathrm{eq}}=2^{1/6}\sigma$ is set to coincide with the zero force distance of the potential $\phi_{\mathrm{rep}}\left(r_{ij}\right)$ of Equation (3). Figure 3 displays the various potential contributions to the total interaction potential for a particle pair. The modified Lennard–Jones potential is shown in blue, with the cut-off tail shown as a dotted line. Likewise the quadratic potential is shown in red. The distance at which the spring elements fail, $r_{cut}$ is marked by the vertical dotted black line. At $r_{ij}>r_{\mathrm{eq}}$, the bonded particle pair experiences a cohesive force due to the spring potential. At $r_{ij}<r_{\mathrm{eq}}$, the particles interaction is governed by the Lennard-Jones potential. At $r_{ij}=r_{\mathrm{eq}}$ all forces are zero.

With the simplified model that we present in this paper, we deliberately exclude dissipation caused by friction or damping. However, some energy is removed from the system when failure of the material occurs. When the distance between two bonded particles exceeds a certain distance $r_{cut}$, the bond is considered to be broken and is removed. The two particles however, may continue to interact with each other or any other particle via the contact potential, and with other particles to which they may still be bonded.

With the three material parameters ϵ, κ, and $r_{cut}$ we have developed a simple model with a minimal number of material parameters with the goal of exploring the potential of DEM for HVI simulations as a proof of principle. The essential parameters are ϵ representing resistance to pressure, κ representing cohesive forces, and $r_{cut}$ representing microscopic failure.

### 2.3. Sizing and Convergence Properties of Our Particle Model

For a simulation to be robust it must reproduce the same results regardless of the arbitrarily chosen number of particles used. This requirement can be separated into two parts: geometric sizing which relates to the value of the model parameters, and numerical convergence which relates to the number of particles used in the simulation. We treat these requirements in turn with the following two case studies.

#### 2.3.1. Geometric Sizing Properties

Geometric sizing is achieved in our modeling approach by defining length invariant sizing parameters γ and λ, obtained from ε and κ by dividing by the length scale σ such that:

(5) $$\begin{array}{cc} \gamma & =\frac{\varepsilon }{\sigma }\;, \end{array}$$

(6) $$\begin{array}{cc} \lambda & =\frac{\kappa }{\sigma }\;. \end{array}$$

Equations (5) and (6) can be solved for ε and κ and substituted into the potential Equations (3) and (4). In this way the simulation results are independent of the model sizes or particle numbers, respectively. We prove this analytically and numerically by considering two models, *A* and *B*, representing the same macroscopic solid of length *L* but with a different number of particles $N_{A}$ and $N_{B}$, scaled by factor *a*, as shown in Figure 2, such that:

(7) $$\begin{array}{cc} N_{B} & =\frac{N_{A}}{a^{3}}\;, \end{array}$$

(8) $$\begin{array}{cc} \sigma_{B} & =a\;\sigma_{A}\;, \end{array}$$

(9) $$\begin{array}{cc} r_{ij_{B}} & =a\;r_{ij_{A}}\;, \end{array}$$

(10) $$\begin{array}{cc} r_{{\mathrm{eq}}_{B}} & =a\;r_{{\mathrm{eq}}_{A}}\;, \end{array}$$

(11) $$\begin{array}{cc} r_{{\mathrm{cut}}_{B}} & =a\;r_{{\mathrm{cut}}_{A}}\;. \end{array}$$

We show that the total potential energy $\Phi_{\mathrm{tot}}=\Phi_{\mathrm{rep}}+\Phi_{\mathrm{coh}}$ of either model is independent of the number of particles *N* used in the simulation. Assuming that every particle $r_{ij}<r_{cut}$ is bonded, the total potential energy of mode *A* can be written as:

(12) $$\begin{array}{cc} \Phi_{tot}\left(A\right) & =\frac{1}{2}\sum_{i,j}^{N_{A}}\Psi \left(A\right)\\ & =\frac{1}{2}\sum_{i,j}^{N_{A}}[\sigma_{A}\lambda {\left(r_{ij_{A}}-r_{eq_{A}}\right)}^{2}\left(H\left(r_{ij_{A}}-r_{eq_{A}}\right)-H\left(r_{ij_{A}}-r_{cut_{A}}\right)\right)+\\ & \;\;\sigma_{A}^{3}\gamma \left\{{\left(\frac{\sigma_{A}}{r_{ij_{A}}}\right)}^{12}-{\left(\frac{\sigma_{A}}{r_{ij_{A}}}\right)}^{6}+1\right\}H\left(r_{eq_{A}}-r_{ij_{A}}\right)]\;, \end{array}$$

where H(x) is the Heaviside step function. The summation is a double summation over *i* and *j*, and the factor $\frac{1}{2}$ corrects the repeated elements in the summation. Similarly, for model *B* we obtain:

(13) $$\begin{array}{cc} \Phi_{tot}\left(B\right)= & \frac{1}{2}\sum_{i,j}^{N_{B}}\Psi \left(B\right)\\ & =\frac{1}{2}\sum_{i,j}^{N_{B}}[\sigma_{B}\lambda {\left(r_{ij_{B}}-r_{eq_{B}}\right)}^{2}\left(H\left(r_{ij_{B}}-r_{eq_{B}}\right)-H\left(r_{ij_{B}}-r_{cut_{B}}\right)\right)+\\ & \;\;\sigma_{B}^{3}\gamma \left\{{\left(\frac{\sigma_{B}}{r_{ij_{B}}}\right)}^{12}-{\left(\frac{\sigma_{B}}{r_{ij_{B}}}\right)}^{6}+1\right\}H\left(r_{eq_{B}}-r_{ij_{B}}\right)]\;. \end{array}$$

Replacing the *B* model size variables in Equation (13) with Equations (7) to (11),

(14) $$\begin{array}{cc} \Phi_{tot}\left(B\right) & =\frac{1}{2}\sum_{i,j}^{N_{B}}[a\sigma_{A}\lambda {\left(ar_{ij_{A}}-ar_{eq_{A}}\right)}^{2}\left(H\left(r_{ij_{A}}-r_{eq_{A}}\right)-H\left(r_{ij_{A}}-r_{cut_{A}}\right)\right)+\\ & \;\;a^{3}\sigma_{A}^{3}\gamma \left\{{\left(\frac{a\sigma_{A}}{ar_{ij_{A}}}\right)}^{12}-{\left(\frac{a\sigma_{A}}{ar_{ij_{A}}}\right)}^{6}+1\right\}H\left(r_{eq_{A}}-r_{ij_{A}}\right)]\\ & =\frac{1}{2}a^{3}\sum_{i,j}^{N_{B}}[\sigma_{A}\lambda {\left(r_{ij_{A}}-r_{eq_{A}}\right)}^{2}\left(H\left(r_{ij_{A}}-r_{eq_{A}}\right)-H\left(r_{ij_{A}}-r_{cut_{A}}\right)\right)+\\ & \;\;\sigma_{A}^{3}\gamma \left\{{\left(\frac{\sigma_{A}}{r_{ij_{A}}}\right)}^{12}-{\left(\frac{\sigma_{A}}{r_{ij_{A}}}\right)}^{6}+1\right\}H\left(r_{eq_{A}}-r_{ij_{A}}\right)]\\ \Phi_{tot}\left(B\right) & =\frac{1}{2}a^{3}\sum_{i,j}^{N_{B}}\Psi \left(A\right)\;. \end{array}$$

Using the fact that the average energy of each particle pair can be found by dividing the total potential by the number of particles, we sum over $N_{A}$ and $N_{B}$ particles in system A to get the same average energy such that

(15) $$\frac{\sum_{i,j}^{N_{B}}\Psi \left(A\right)}{N_{B}}=\frac{\sum_{i,j}^{N_{A}}\Psi \left(A\right)}{N_{A}}\;.$$

Equations (12) and (14) can then be substituted into Equation (15):

(16) $$\frac{\Phi_{tot}\left(B\right)}{\frac{1}{2}a^{3}N_{B}}=\frac{\Phi_{tot}\left(A\right)}{\frac{1}{2}N_{A}}\;,$$

(17) $$\Phi_{tot}\left(B\right)=\frac{a^{3}N_{B}\Phi_{tot}\left(A\right)}{N_{A}}\;.$$

Finally, Equation (7) can be substituted into Equation (17):

(18) $$\Phi_{tot}\left(B\right)=\frac{a^{3}N_{B}\Phi_{tot}\left(A\right)}{a^{3}N_{B}}$$

(19) $$=\Phi_{tot}\left(A\right)\;,$$

resulting in the same total potential energy independent of the number of particles. For the kinetic energy of the system to be independent of size, the mass of the particles must also be scaled. This is done by solving

(20) M=mN

for the individual particle mass *m*, where *M* is the total mass and *N* is the number of particles in the system.

**Figure 4:**
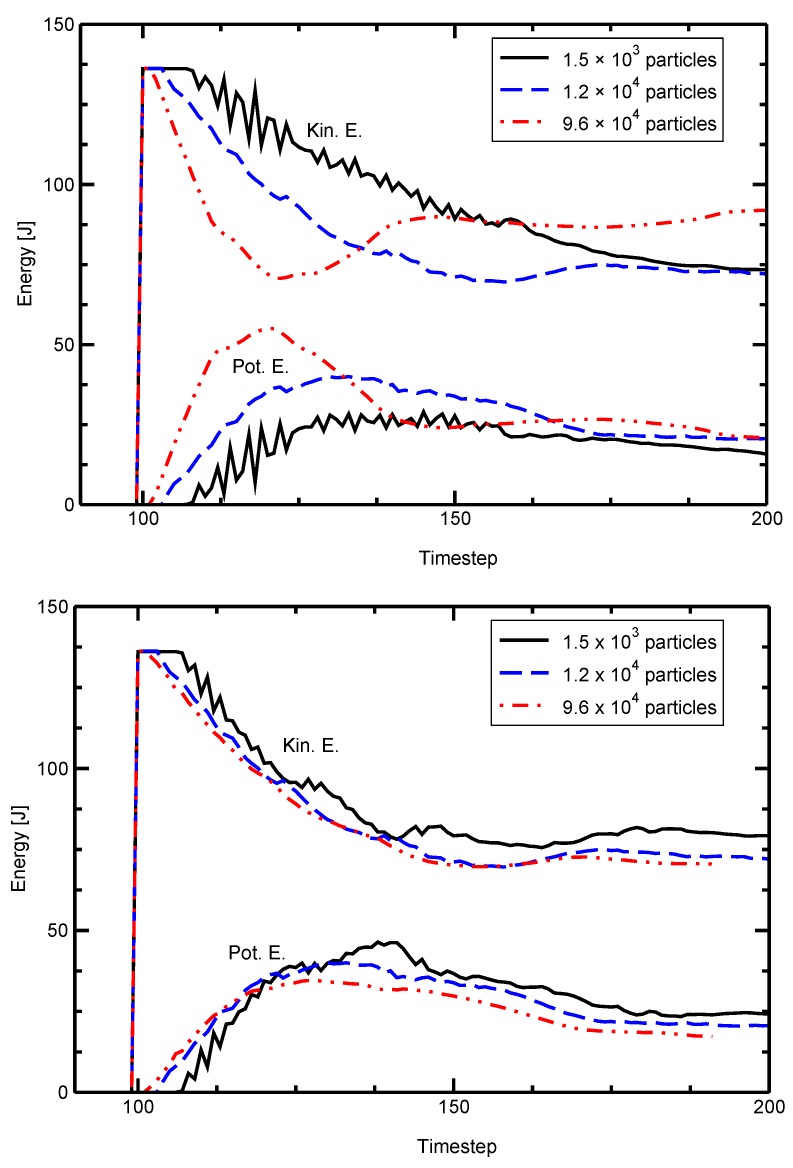
Total potential and kinetic energies of colliding plates with different numbers of particles. (**top**) The unscaled simulation is strongly affected by the number of simulation particles used. (**bottom**) Resizing the simulation renders the resulting energy independent of the number of particles used.

The model’s sizing behavior is demonstrated by evaluating the potential and kinetic energies of two colliding plates. The same geometry is modeled with different numbers of particles. Figure 4 shows the scaled and unscaled effects on the energy in the plates with the number of particles spanning almost three orders of magnitude. The unscaled simulations are widely divergent while the geometric sizing renders the three solutions very similar.

#### 2.3.2. Convergence Properties

**Figure 5:**
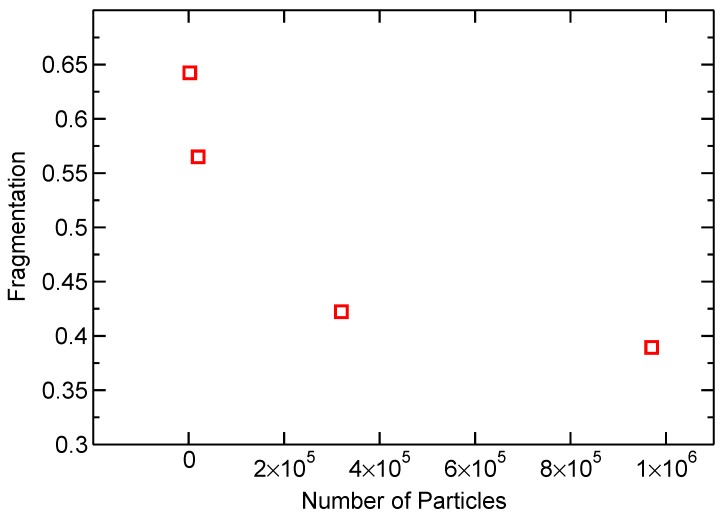
Fragmentation in debris clouds simulated with a varying number of particles shows convergence.

Figure 5 shows a convergence study measuring the fragmentation of the debris cloud after HVI. In this paper, a fragment is defined as an interconnected collection of simulation particles; the smallest fragment consists of a single particle. Fragmentation is defined as the ratio of fragments over the total number of simulation particles. Therefore, a fragmentation equal to one represents a simulation where every single bond is broken, and therefore the number of fragments is equal to the number of simulation particles. In Figure 5, each simulation performed was first sized geometrically, therefore representing the same physical system, only with a different number of particles.

The study shows that the simulations begin to converge at about a quarter of a million particles. Simulations for calibrating the model parameters, described in Section 3.1, were performed with number of simulation particles $N=2.7\times 10^{5}$. The number of simulation particles used was increased to $N=7.4\times 10^{5}$ particles for the final simulations. A stable timestep for the simulations was found to be $\Delta t=1\times 10^{-10}$ s.

## 3. Results and Discussion

In this section we discuss the results of our simulation study. After a detailed analysis of our choice of model parameters, we validate our simulations by comparison with experiments. This is followed by a comprehensive parameter study of HVI simulations which we use to analyze the shape of the resulting debris cloud and the degree of material fragmentation after impact.

### 3.1. Choice of Model Parameters

**Figure 6:**
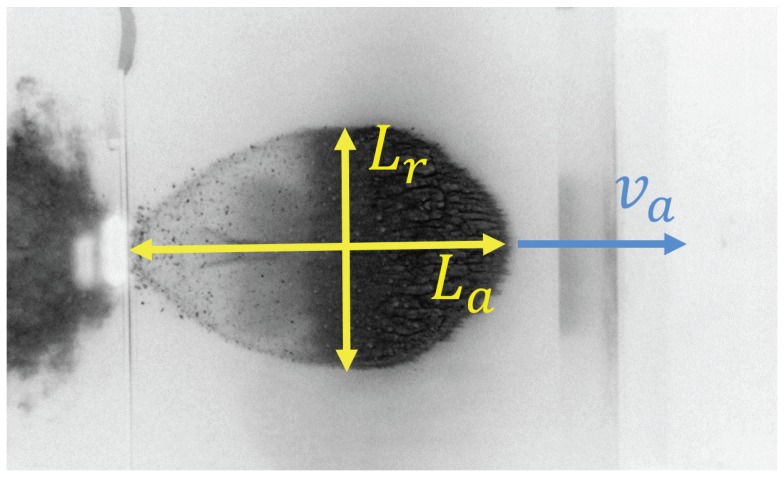
Experimental high-speed photograph of the debris cloud showing the cloud’s length ratio $R=L_{a}/L_{r}$, and axial expansion velocity, $v_{a}$. The witness plate seen in the right side of the snapshot is shown in Figure 8. The image intensity has been inverted for better viewing. Figure courtesy of Fraunhofer Ernst–Mach–Institut.

The three free parameters of our model, ε,κ, and $r_{cut}$, are empirically fit by comparing the simulation results directly to a recent experiment involving aluminum spheres impacting aluminum plates. We used an experiment previously performed at our institute with an impact velocity $v_{0}=6.5$ ${\mathrm{kms}}^{-1}$ and t/D=0.41. Figure 6 shows a high-speed camera image of the experiment with the image’s intensity inverted to allow for better viewing. Due to the challenges in performing HVI experiments, multiple experiments with the exact same parameters were unavailable. The experiment shown in Figure 6 was taken from a series of experiments studying the scalability of HVI, all of which had the same cloud expansion properties. This gives us some degree of confidence that the values measured from this single experiment are representative of HVI phenomena and therefore valid for fitting our model’s parameters.

**Figure 7:**
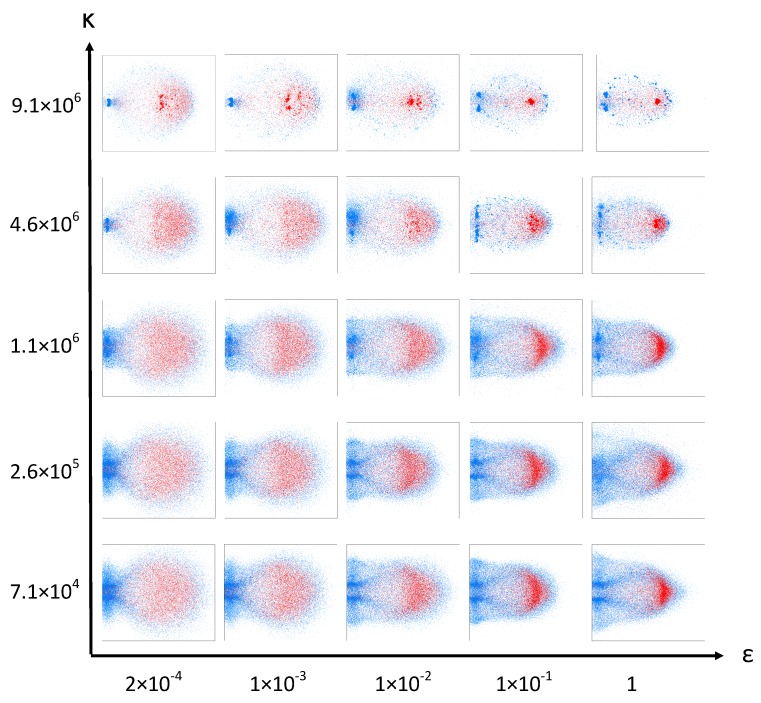
Simulation snapshots of the debris cloud calculated with different values of ε and κ taken at 32 μs after impact. Impactor particles are shown in red and target particles are shown in blue.

Figure 7 shows simulation snapshots of the resulting debris cloud caused by a HVI with $v_{0}=6.7$ ${\mathrm{kms}}^{-1}$ and t/D=0.425, organized into the relevant range of the (ε,κ)-parameter space. The snapshots are full 3D simulations displayed from a side view. Parameter $r_{cut}$ is set to 1.5σ for this series of simulations. Parameter κ is the spring constant in the linear cohesive spring force of Equation (4). This parameter models the cohesive force of the material. Small values of κ lead to a very weak material that fragments almost completely, while large values result in stronger cohesive forces between the elements and thus a much stronger material that resists fragmentation.

The parameter ε scales the strength of the repulsive Lennard-Jones potential, influencing the stiffness of the spherical elements. A low value of ε allows the spheres to overlap each other to a higher degree (soft spheres). The overlapping allows the high-velocity impactor particles to more easily penetrate past the stationary target particles without transferring as much momentum. Only a fraction of the impactor particles fully collide with the stationary target particles and transfer their momentum. Because only some particles in the impactor directly impact the target particles, while others penetrate the target without transferring much momentum, more of the connecting bonds are broken leading to smaller fragment clusters in the debris cloud. The end effect, which can be seen at low values of ε in Figure 7, is more spread in the debris cloud as some particles continue their trajectory with little change in velocity while others loose almost all of their momentum during impact.

At high values of ε, the impactor particles cannot interpenetrate as much and therefore transfer their momentum to the target particles more evenly and less comminution occurs. This is apparent at large values of κ in Figure 7 where we can see that a smaller ε results in more fragmentation while larger values of ε retain an unfractured central chunk. Interestingly, large values of ε cause the shock wave to travel further and faster, hence causing more damage in the plate.

On the extreme top right corner of the chosen parameter space of our model displayed in Figure 7, the material is too hard and tough to represent the experimental findings displayed in Figure 6. On the lower left extreme, the material modeled is too weak and brittle. Consequently, we conclude that the best fitting parameters ε and κ to represent the experimental findings lie somewhere within the parameter space.

For finally determining the simulation parameters that best fit to the experiment, we use a visual, qualitative comparison and two quantitative properties. These are: the degree of fragmentation, the debris cloud’s length ratio $R=L_{a}/L_{r}$, and the axial cloud expansion velocity $v_{a}$, as seen in Figure 6.

**Figure 8:**
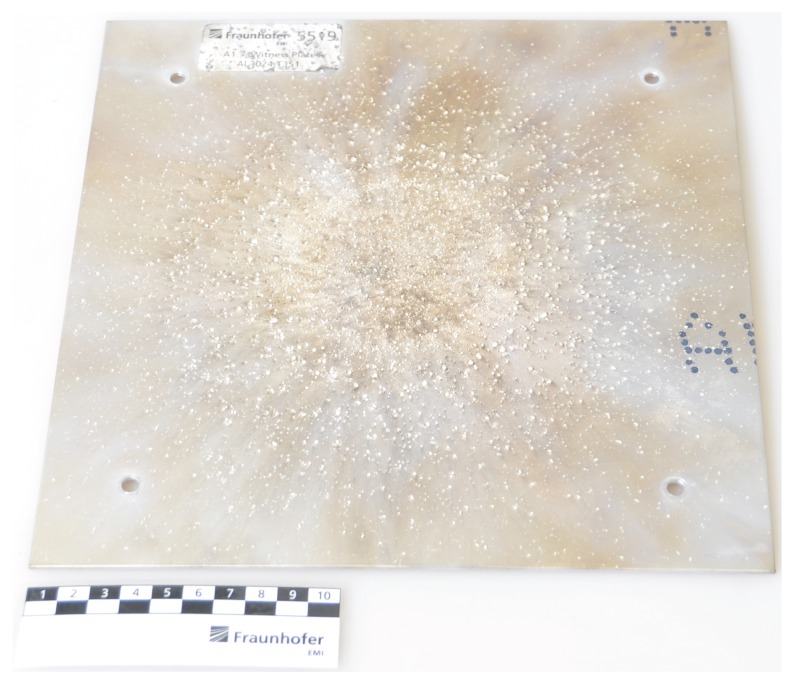
The witness plate from the experiment shown in Figure 6 contains many small impact craters left by the debris cloud fragments. The average crater has a diameter of approximately 1 mm, but there is no crater left by a large central fragment. Figure courtesy of Fraunhofer Ernst–Mach–Institut.

A close examination of the experimental situation shown in Figure 6 reveals the abundance of small fragments of different sizes in the cloud. Small impact craters on a witness plate, as shown in Figure 8, confirm that the debris cloud contains many small and medium fragments of approximately 1 mm, and that no larger pieces of the impactor remain intact. Examining the debris cloud simulation snapshots in Figure 7 reveals that all the simulations with $\kappa \leq 1.14\times 10^{6}$ exhibit almost complete fragmentation with very small fragments on the order of 0.1 mm. This does not match the experimental evidence left on the witness plate in Figure 8, allowing us to eliminate this range of κ as possible parameters.

The parameters resulting in simulations with a large central fragment, such as (ε=1, $\kappa =9.12\times 10^{6}$) and ($\varepsilon =0.1,\;\kappa =9.12\times 10^{6}$) in Figure 7, can also be ruled out because of the lack of a large crater in the witness plate.

**Figure 9:**
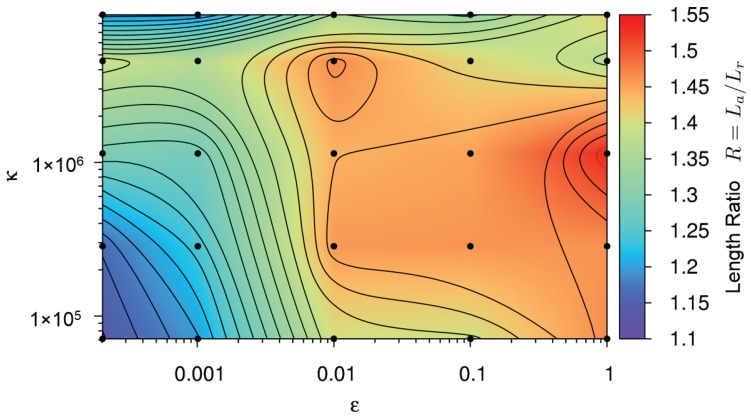
Contour plot representing the debris cloud’s axial to radial extension ratio, $R=L_{a}/L_{r}$, for each simulation in Figure 7. Color code refers to the length ratio *R* shown on the right side of the figure. See main text for details.

As a second parameter, the dimensionless length ratio *R* of the cloud provides a quantitative measure of the cloud shape. A ratio equal to one represents a circular shape of the debris cloud and values larger than one represents an elliptical shape of the cloud. The target value, as measured in the experiment presented in Figure 6, is 1.55. In our quantitative analysis, the length ratio *R* is calculated for each pair of (ε, κ), shown as black dots in Figure 9, with the values interpolated over the parameter space to create a contour plot.

In Figure 9, we can see a plateau at a length ratio of approximately 1.47 and a peak of 1.54. Although the simulation point ($\varepsilon =1,\;\kappa =1.14\times 10^{6}$) with a shape ratio of 1.54 is the closest to the experimental value of 1.55, it was previously ruled out when examining the degree of fragmentation, along with most of the other points in the plateau. This leaves the local maximum at (ε=0.01, $\kappa =4.56\times 10^{6}$) as the next best option.

**Figure 10:**
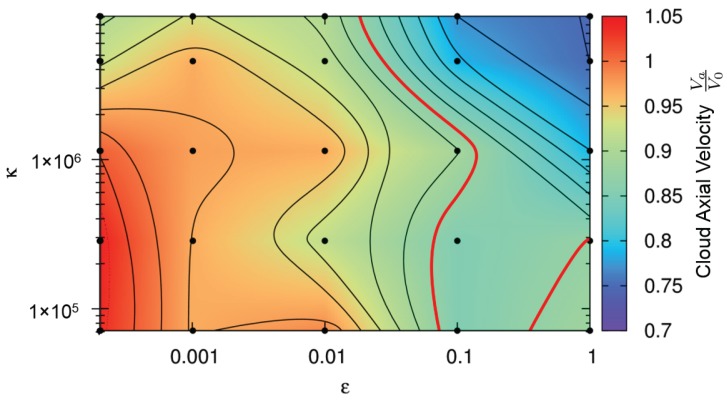
Contour plot representing the debris cloud’s axial expansion velocity normalized by the impact velocity. Color code refers to the velocity $v_{a}/v_{0}$ shown on the right side of the figure. The experimentally measured value is shown as a red contour line. See main text for details.

Our third quantitative comparison criteria is the cloud expansion velocity $v_{a}$, which we normalize with the initial impact velocity $v_{0}$. A contour plot of this quantity is shown in Figure 10, along with the corresponding values of ε and κ. Experimentally, the cloud expansion velocity is determined by measuring the distance traveled by the cloud front between two high-speed photographs and dividing by the time between each snapshot. This can be done because after impact, the cloud continues on its trajectory at constant velocity. The red contour in Figure 10 represents the experimentally measured axial velocity. The axial velocities measured from the simulations, shown in Figure 10, exhibit a decreasing trend with increasing ε and only small variations in velocity depending on κ. The simulation points closest to the red line which have not already been ruled out by the other first two comparison criteria remain as the best choices for a realistic representation of the experiment.

**Figure 11:**
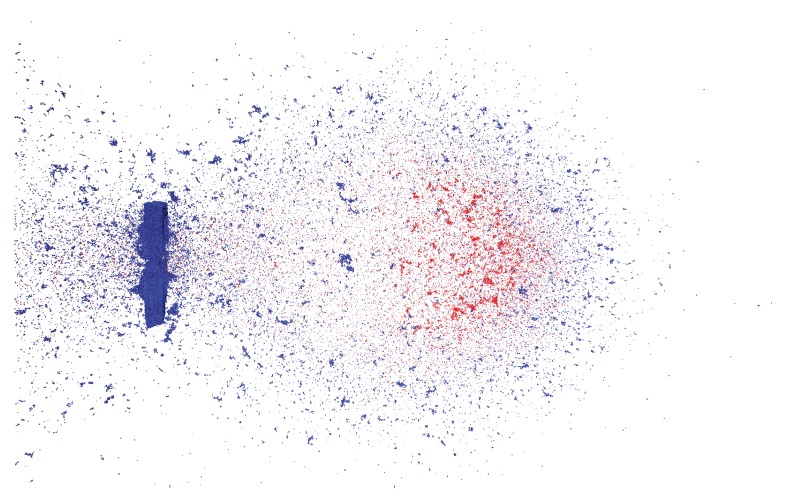
A snapshots of the resulting 3D simulation using the best fit parameters: ε=0.01, $\kappa =4.56\times 10^{6}$ and $r_{cut}=1.5\sigma$. This simulation corresponds to the experiment shown in Figure 6. Impactor particles are in red and target particles in blue.

**Figure 12:**
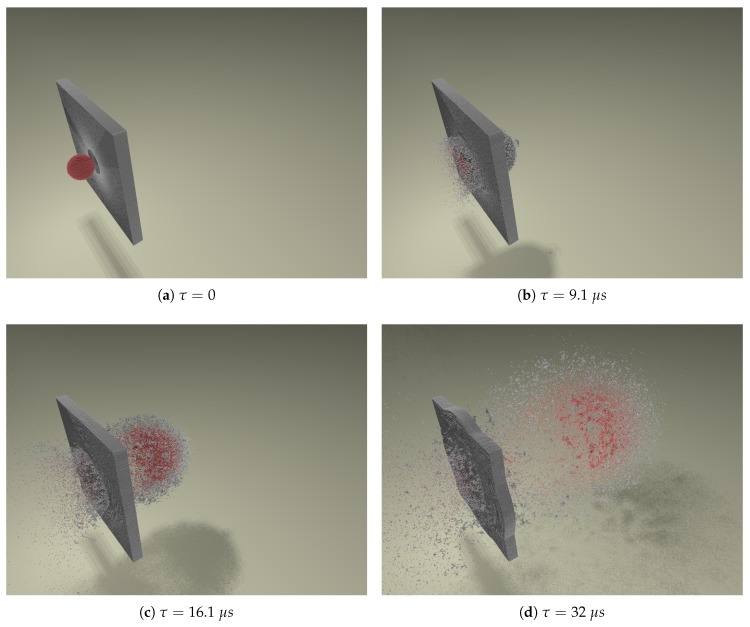
A series of 3D simulation snapshots showing an aluminum sphere impacting an aluminum plate at hypervelocity. Red indicates impactor particles and gray indicates target plate particles. The simulation was run with $N=7.4\times 10^{5}$ particles, with a timestep of $\Delta t=1\times 10^{-10}$ s, and took nine hours to complete on a single processor. Impact parameters are: $v_{0}=6.7\;{\mathrm{kms}}^{-1}$ and t/D=0.425. A video sequence of this simulation can be found as Supplementary Material in Video S1.

Putting everything together, we choose the parameters ε=0.01, $\kappa =4.56\times 10^{6}$, and $r_{cut}=1.5\sigma$ to be the closest match to the experiment. Figure 11 shows a simulation snapshot of the debris cloud 32 μs after impact with this choice of model parameters. Blue represents target plate particles and red represents impactor particles. Comparing this figure directly to the experimental photograph in Figure 6, shows the similarity between experiment and simulation. Figure 12 shows a 3D series of snapshots of the developing debris cloud directly after impact. Red particles represent the impactor and gray particles represent the target. A video sequence of this simulation can be found as Supplementary Material in Video S1.

### 3.2. Validation with Experiment

We perform numerous HVI simulations at a variety of different impact velocities and t/D ratios and compare them to the corresponding experiments. One challenge is the limited quantifiable data which can be obtained from HVI experiments. The extremely short time scale and limited instrumentation mean that often high-speed photographs are the only data available from the experiments. This restricts the quantitative comparison possible between our proposed numerical model and experiments. Nevertheless, a comparison is performed from the data that are available.

#### 3.2.1. Extension of Debris Cloud

One of the measurable quantities from the experiments is the debris cloud’s expansion velocity. Normalizing the expansion velocities with the impact velocity, $v_{a}/v_{0}$, allows us to meaningfully compare cloud characteristics even at different impact velocities.

**Figure 13:**
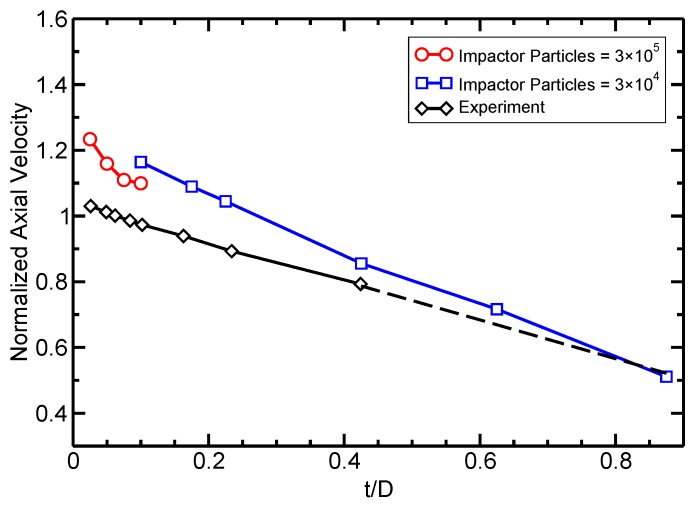
Debris cloud axial expansion velocity with $v_{0}=6.7\;{\mathrm{kms}}^{-1}$ at different t/D ratios. The simulation results are compared to experiments by Piekutowski [50]. The dotted line represents linearly extrapolated experimental data.

In Figure 13, we compare the calculated debris cloud’s axial expansion velocity with experimental values performed by Piekutowski [50] at an impact velocity of 6.7 ${\mathrm{kms}}^{-1}$ with varying t/D ratio. The diameter of the impacting sphere was 9.53 mm in the simulation and experiment. At larger t/D ratios, the simulation model had to be rescaled to avoid simulating an unreasonable number of particles as the plate thickness increased. The dotted line represents linearly extrapolated experimental data.

In Figure 13, the simulation over-predicts the expansion velocities, but still captures the overall decreasing trend. This decreasing trend is due to the increase in thickness of the target plate at higher t/D ratios. Since the sphere’s size remains constant, a thicker plate requires more momentum to be transferred from the impactor particles to the plate particles. This increases the total mass in the debris cloud, but reduces its velocity.

The simulations’ over-prediction of expansion velocities, as seen in Figure 13, result from the lack of a dissipative energy term in our model. Physically speaking, the passing of a shock wave is a highly transient process during which some of the kinetic energy is converted into heat as the material behind a shock wave experiences a sudden jump in thermodynamic variables such as pressure, energy, and density. This jump between two points of the Hugoniot curve takes place along the Rayleigh line and is a highly nonisentropic process. The rarefaction waves that bring the material back to ambient condition occur on an isentropic path. The difference in entropy gained in the process is therefore converted into heat which is absorbed by the material. If the shock pressure is high enough, melting or vaporization will occur. Bjork and Olshaker [51] analytically estimated the impact velocities at which high enough shock pressures are generated to cause incipient and complete melting in aluminum. Their results, shown in Table 1, indicate that a certain degree of melting occurs in the experiments.

**Figure 14:**
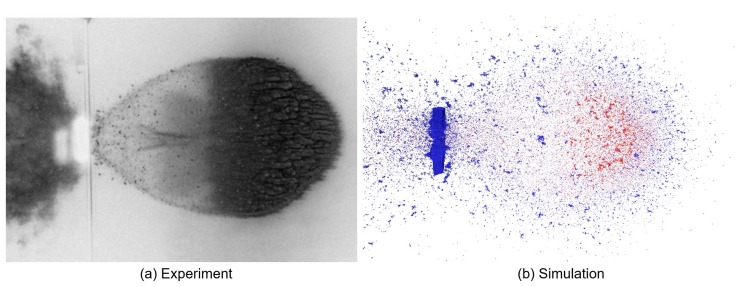
Simulations with high t/D ratios match the experiment closely with complete fragmentation of the impactor and a similar cloud shape. (**a**) High-speed photograph of experiment (intensity inverted); (**b**) 3D simulation shown with $v_{0}=6.7\;{\mathrm{kms}}^{-1}$ and t/D=0.425.

Without any dissipative effects in the model to account for heating and melting, all of the energy from the passing shock wave, except what is lost within the broken bonds, is recovered and transformed into kinetic and potential energy. This results in too much kinetic energy assigned to certain particles, leading to an overestimate of the cloud expansion velocity when compared to the experiment. A secondary effect of the lack of energy dissipation is a more diffuse boundary in the simulation debris cloud caused by a large variation in particle velocities. In contrast, the heating and melting in the experiment limit the particle velocities and help to create a sharper cloud boundary, as can be seen in Figure 14.

#### 3.2.2. Shape and Degree of Fragmentation

**Figure 15:**
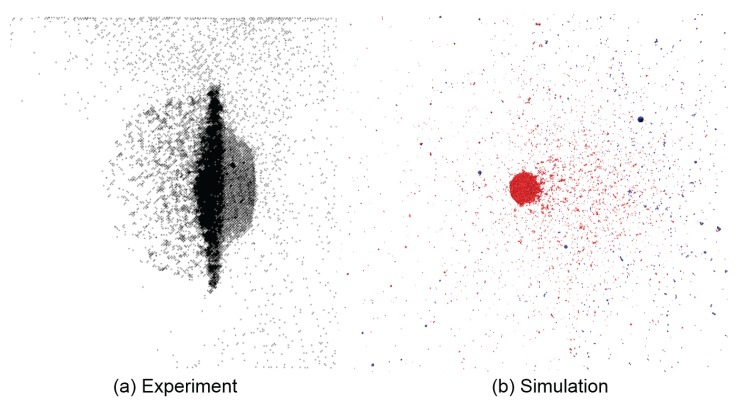
The shape of the debris cloud is highly affected by the t/D ratio. (**a**) High-speed radiograph of experiment; (**b**) 3D simulation shown with $v_{0}=6.7\;{\mathrm{kms}}^{-1}$ and t/D=0.05. The simulation performs poorly at extremely small t/D ratios, but very well at large values, cf. Figure 14. Experimental snapshot from [50].

Although the expansion velocities of the debris cloud provide useful and easily quantifiable information, they do not completely characterize the debris cloud; namely, the shape and degree of fragmentation of the cloud is not accounted for. Unfortunately, experiments do not generally provide a quantitative analysis of the fragmentation of the debris cloud distribution, so one usually depends on visual inspection. We provide such a visual comparison in Figure 14 and Figure 15, which show simulation and experimental snapshots of the debris clouds resulting from the impact of an aluminum sphere on plates of different thicknesses at $v_{0}\approx 6.7\;{\mathrm{kms}}^{-1}$. When the equivalent simulation and experiments are compared, it becomes apparent that the shape and degree of fragmentation play an important role in the debris cloud characterization.

Similarities in debris cloud shape and fragmentation level can be seen in Figure 14 showing impacts with high t/D ratios, but strong differences in shape and fragmentation occur at the low t/D ratio range. Figure 15 compares the experiment and simulation debris cloud resulting from an impact with t/D=0.05, which exhibit very noticeable differences such as:

- The well defined front end (left side of debris cloud) as seen in the experiment is missing in the simulation.
- The large central fragment in the simulation did not fracture into a distinctive debris bubble behind the dense cloud center (right side of debris cloud) as seen in the experiment.

The lack of a well defined front end structure is due to the absence of dissipative mechanisms in the model to account for heating and melting as previously explained. The failure to form a distinctive debris bubble at the rear of the cloud results from the model’s limitation when the shock pressures are too low. The amplitude of a shock wave in HVI is dependent on the impact velocity and the combined geometry of target and impactor.

Upon impact, two shock waves form and propagate away from the interface between the plate and impacting sphere. When these shock wave reach the free back end of the plate or impacting sphere they are reflected as rarefaction waves, which are tensile waves. If the net tensile stress due to any rarefaction wave exceeds the fracture stress of the material, spallation will occur.

Because rarefaction waves propagate faster than shock waves, at small ratios of plate thickness to projectile length, i.e., the t/D ratio, the rarefaction wave reflected off the target plate may overtake and attenuate the shock wave in the projectile. Therefore in HVI with a low t/D ratio, the impactor may only experience a weakened compressive wave. In such cases, the remaining amplitude of the compressive and tensile stresses in the impactor may no longer be many times higher than the material’s shear and tensile strength. In such a case, our original assumption of material strength playing a very small role in the overall system behavior no longer holds true and the model ceases to yield accurate results.

### 3.3. Analyzing Fragmentation

In light of our model’s results depending on the shock impact pressure level, we define the conditions under which strong enough shocks occur for our model to accurately reproduce the experiments. One way of indirectly determining the strength of the shock wave in the impactor and target is the degree of fragmentation. If the projectile has undergone a high degree of fragmentation, the pressure wave must have had a large amplitude, in which case material strength plays only a minor role and we can expect the model to accurately mimic the experimental situation. Maximum fragmentation should occur at the t/D ratio where the shock wave in the projectile reaches the back end of the sphere before the rarefaction wave reflected off the free end of the target plate can overtake it. At larger ratios, the shock strength in both the target and impactor will decrease due to geometric damping. Naturally the impact velocity also plays a very important role in the fragmentation with higher velocities producing stronger pressure amplitudes than lower velocities.

Therefore, using fragmentation as a measure of the validity of our model, we quantify fragmentation *F* as

(21) $$F=\frac{N_{\mathrm{frag}}}{N_{\mathrm{part}}}\;;$$

where $N_{\mathrm{part}}$ is the number of simulation particles in the debris cloud and $N_{\mathrm{frag}}$ is the number of fragments in the debris cloud.

**Figure 16:**
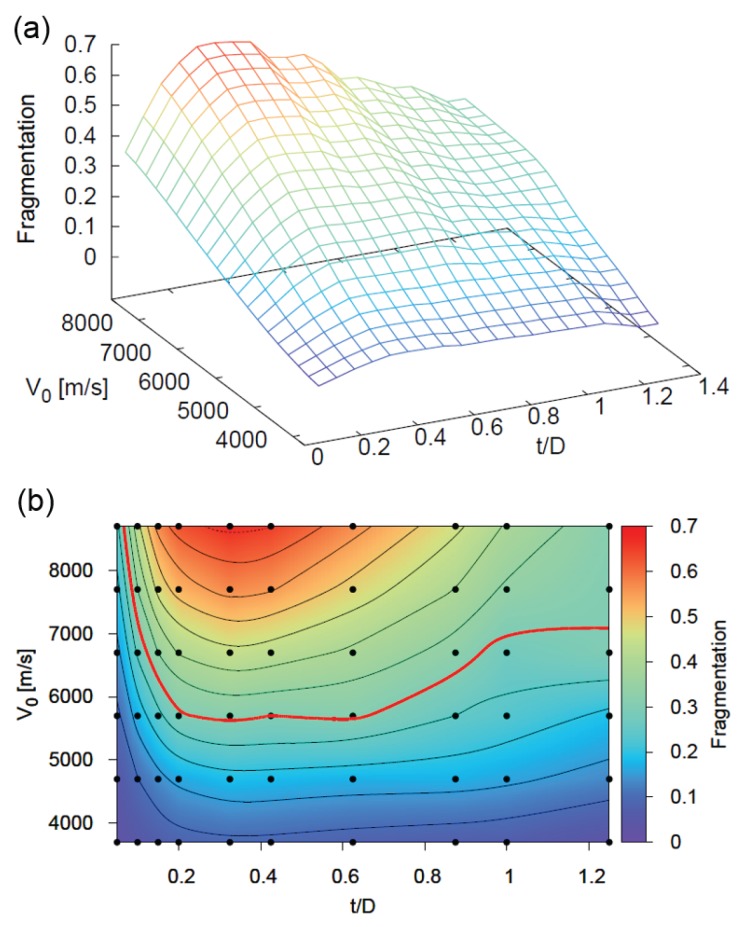
Fragmentation of the debris cloud at various t/D ratios and impact velocities $v_{0}$. (**a**) Three–dimensional representation of fragmentation level; (**b**) Two–dimensional contour plot of fragmentation level. Black dots represent simulation points from which the contour surface was created and the red line is the fragmentation cutoff boundary.

We apply the definition of fragmentation from Equation (21) to perform a comprehensive parameter study varying t/D and $v_{0}$, shown in Figure 16. Plots (a) and (b) in Figure 16 both display the same fragmentation information in 3D and 2D respectively. The lines in Figure 16b are contour lines of constant fragmentation. As expected, fragmentation increases monotonically with velocity because of the higher shock pressures. Also, as predicted, there is a local maximum for the t/D ratio.

With the fragmentation information available, we need only to choose a cutoff value below which the material strength begins to play a dominant role. Below this fragmentation value, the impact conditions violate the original assumptions made when formulating the model. Above this fragmentation value, our model accurately captures the physical phenomena.

**Figure 17:**
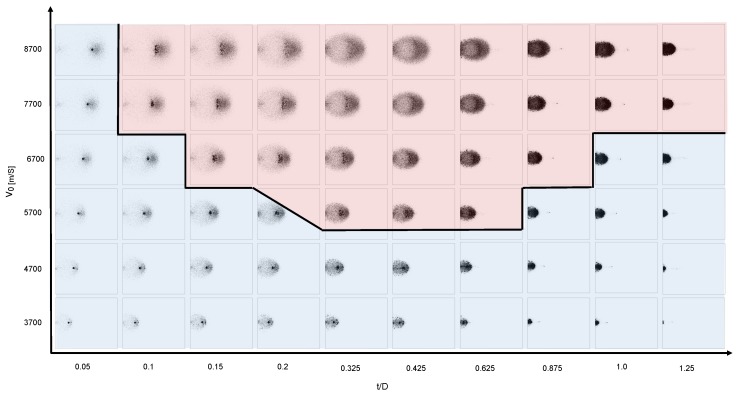
Snapshots of the debris clouds produced under different impact conditions. The impact conditions leading to a valid model is shown in red; invalid regions are shown in blue.

Lacking a quantitative way of determining the exact fragmentation value to set as the cutoff, we used an iterative process of comparing the simulation snapshots in Figure 17 and the contour lines of constant fragmentation in Figure 16b to qualitatively judge the cutoff boundary. Particular attention was given to the size of the large central fragment when choosing the cutoff value. Using this method, we define fragmentation F=0.3 to be the cutoff value. The contour with this value is shown in red in Figure 16b and Figure 17 provides an overview of the debris cloud under different impact conditions. The figure depicts the region where our model is valid, shown in red, and the region that does not correspond to the experiments, shaded in blue.

## 4. Conclusions

In this paper, we explore the suitability of simulating impacts at velocities beyond 5 ${\mathrm{kms}}^{-1}$ with DEM. We propose a very simple model with three free parameters using two cohesive and repulsive potentials. In developing the model, we postulated that the extremely high pressures experienced by the material under HVI would relegate its material strength to a minor role. We assume that the material under impact behaves like a viscous fluid instead of a rigid solid, hence allowing a simplified model.

The model’s parameters are determined by comparing the simulation results to experimental data taken from literature and performed at the Fraunhofer Ernst–Mach–Institute’s hypervelocity testing facility. When evaluating the model’s suitability, we find good correspondence between simulation and experiment when the impact conditions lead to strong shock waves propagating through the material, but poor results when the impact velocity or geometry hinders strong shocks from forming. We present here a comprehensive parameter study to evaluate the model’s range of validity, in terms of impact velocity and geometry.

In a follow-up study currently underway, we are extending our model to account for dissipative effects such as heating and melting. We plan to investigate more complex and comprehensive models that will lead to accurate simulations at low shock pressures. We are also expanding the model to new impact geometries such as Whipple shields used for spacecraft shielding and to different impactor geometries such as cylinders. We plan to analyze the debris cloud resulting from such impacts with respect to fragment size, kinetic energy, cloud shape, and expansion velocity.

## Figures and Tables

**Table 1:** Approximate shock-melting properties of aluminum [51].

	Shock	Impact
	Pressure [GPa]	Velocity [km/s]
Incipient Melting	70	5.6
Complete Melting	100	7.0

## Supplementary Materials

The following video sequence is available online at www.mdpi.com/1996-1944/10/4/379/s1, Video S1: Hypervelocity Impact on Thin Plate.
